# Multiple-Insecticide Resistance in *Anopheles gambiae* Mosquitoes, Southern Côte d’Ivoire

**DOI:** 10.3201/eid1809.120262

**Published:** 2012-09

**Authors:** Constant V.A. Edi, Benjamin G. Koudou, Christopher M. Jones, David Weetman, Hilary Ranson

**Affiliations:** Liverpool School of Tropical Medicine, Liverpool, UK (C.V.A. Edi, B.G. Koudou, C.M. Jones, D. Weetman, H. Ranson);; Centre Suisse de Recherches Scientifiques en Côte d’Ivoire, Abidjan, Côte d’Ivoire (C.V.A. Edi, B.G. Koudou);; and Université d’Abobo-Adjamé, Abidjan (B.G. Koudou)

**Keywords:** pyrethroids, DDT, organophosphates, carbamates, acetylcholinesterase, Anopheles gambiae, mosquitoes, Côte d’Ivoire, malaria, vector-borne infections, insecticide resistance

## Abstract

Malaria control depends on mosquito susceptibility to insecticides. We tested *Anopheles gambiae* mosquitoes from Côte d’Ivoire for resistance and screened a subset for target site mutations. Mosquitoes were resistant to insecticides of all approved classes. Such complete resistance, which includes exceptionally strong phenotypes, presents a major threat to malaria control.

## Abstract 2

Preventing malaria used to seem as simple as killing the vector, the mosquito; however, a recent study shows that this concept is now anything but simple. The highly effective use of insecticide-treated bed nets and indoor insecticide spraying is being challenged by mosquito resistance to insecticides. In West Africa, populations of this mosquito vector are now resistant to all 4 classes of insecticide approved for this use. And no new classes of insecticide are anticipated until 2020, at the earliest. Development of newer classes of insecticide is crucial because if resistance continues unchecked, the hard-earned progress in malaria control in Africa could be quickly reversed.

Targeting the mosquito vector is the most effective way to prevent malaria transmission; worldwide, this method accounts for more than half of malaria control expenditures (*1*,*2*). During the past decade, increased use of insecticide-treated bed nets and indoor residual spraying have made a pivotal contribution toward decreasing the number of malaria cases (*1*). However, these gains are threatened by the rapid development and spread of insecticide resistance among major malaria vectors in Africa (*3*). To keep vector resistance from undermining control programs, insecticide-resistance management strategies must reduce the current overreliance on pyrethroids. These compounds are used widely for indoor residual spraying and uniquely for insecticide-treated bed nets. However, having a limited number of insecticides available for malaria vector control restricts options for effective insecticide resistance management. Only 4 classes of insecticide, which share 2 modes of action, are approved by the World Health Organization (WHO).

A mutation at a single target site can result in mosquito resistance to DDT and pyrethroids or to organophosphates and carbamates. Furthermore, mosquitoes can express multiple insecticide-resistance mechanisms (*4*). For example, in several populations of the major malaria vector in Africa, *Anopheles gambiae* s.l. mosquitoes, mutations in the DDT/pyrethroid target site, known as knockdown resistance (*kdr*) alleles, have been found in conjunction with resistance alleles of the acetylcholinesterase gene (*Ace-1^R^*), the target site of organophosphates and carbamates (*5*). To date, however, these cases of multiple-insecticide resistance have been restricted by the relatively low prevalence of organophosphate/carbamate resistance and the limited effect that *kdr* mutations alone have on pyrethroid-based interventions (*6*). We report a population of *An. gambiae* mosquitoes from a rice-growing area of southern Côte d’Ivoire that have high frequencies of *kdr* and *Ace-1^R^* alleles and unprecedentedly high levels of phenotypic resistance to all insecticide classes available for malaria control.

## The Study

During May–September 2011, mosquito larvae were collected in irrigated rice fields surrounding Tiassalé, southern Côte d’Ivoire (5°52′47′′N; 4°49′48′′W) and reared to adults in insectaries on a diet of MikroMin (Tetra, Melle, Germany) fish food. A total of 1,571 adult female *An. gambiae* s.l. mosquitoes, 3–5 days of age, were exposed to 1 of 5 insecticides (0.1% bendiocarb, 1.0% fenitrothion, 0.75% permethrin, 0.05% deltamethrin, 4% DDT) or a control papers for 1 hour, according to standard WHO procedures (*7*). Mosquito deaths were recorded 24 hours later. DNA was extracted from individual mosquitoes according to the LIVAK method (*8*), and a subsample of 500 mosquitoes were all found to be the M molecular form of *An. gambiae* s.s. by using the SINE-PCR method (*9*). The target site mutation G119S in the *Ace-1* gene (*Ace-1^R^*) and L1014F and L1014S *kdr* mutations were screened by using restriction fragment length polymorphism (*10*) or TaqMan assays (*11*), respectively.

**Figure 1:**
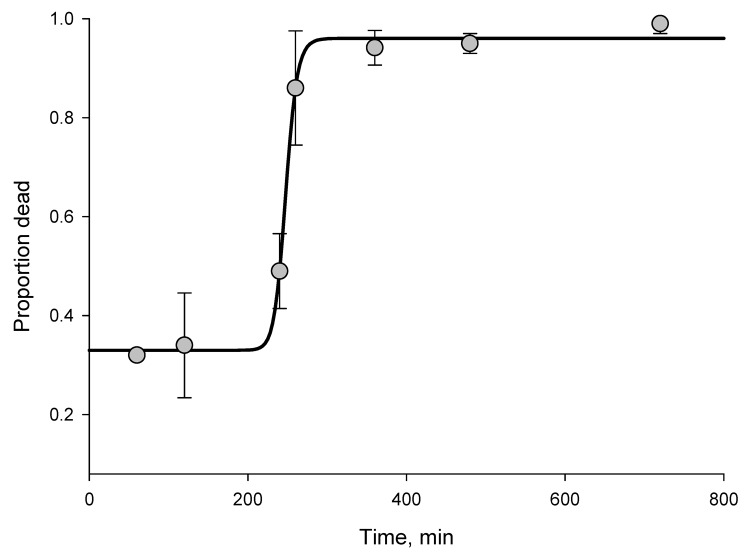
Time-mortality curve for wild-caught *Anopheles gambiae* mosquitoes from Tiassalé, southern Côte d’Ivoire, exposed to deltamethrin (median time to death = 248 minutes). Logistic regression line was fitted to time-response data by using SigmaPlot version 11.0 (www.sigmaplot.com). R^2^ = 0.96. Error bars indicate SEM.

**Figure 2:**
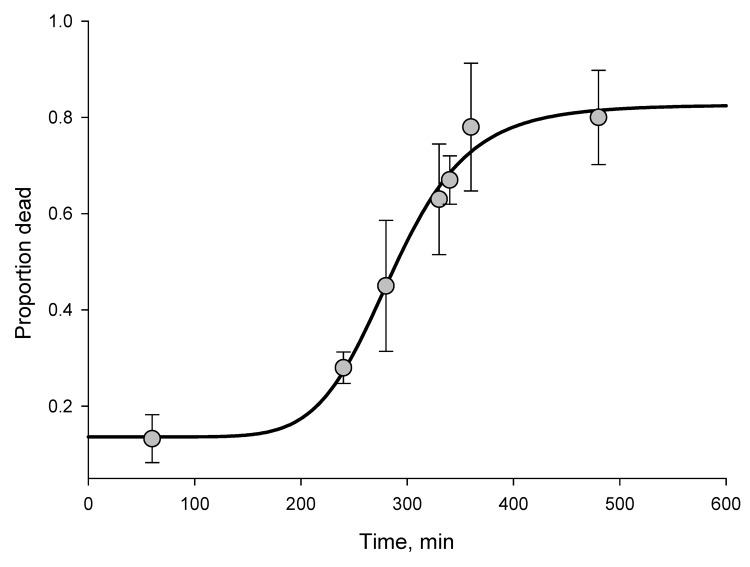
Time-mortality curve for wild-caught *Anopheles gambiae* mosquitoes from Tiassalé, southern Côte d’Ivoire, exposed to bendiocarb (median time to death = 286 minutes). Logistic regression line was fitted to time-response data by using SigmaPlot version 11.0 (www.sigmaplot.com). R^2^ = 0.88. Error bars indicate SEM.

According to WHO criteria, *An. gambiae* mosquitoes from Tiassalé are resistant to all insecticide classes, and resistance is extremely prevalent; more than two thirds of mosquitoes survived the diagnostic dose for 4 of the 5 insecticides tested (Table 1). To assess the level of resistance, we exposed the Tiassalé population and a susceptible laboratory population of *An. gambiae* (Kisumu) mosquitoes to the pyrethroid deltamethrin or the carbamate bendiocarb for a range of exposure times and assessed deaths 24 hours later (Technical Appendix). We found an unexpectedly strong resistance phenotype to the 2 insecticides (Figure 1, Figure 2). For deltamethrin, 4 hours of exposure were required to kill 50% (median lethal time, [LT_50_]); in comparison, the LT_50_ for the susceptible Kisumu strain was <2 minutes (resistance ratio = 138) (Technical Appendix). Similarly, the LT_50_ for bendiocarb was nearly 5 hours for the Tiassalé strain yet <12 minutes for the susceptible strain (resistance ratio = 24) (Technical Appendix).

**Table 1:** Prevalence of insecticide resistance in *Anopheles gambiae* mosquitoes, M form, from Tiassalé, Côte d’Ivoire, 2011

Insecticide	No. tested*	No. dead	% Dead (95% CI)
Permethrin	288	69	24.0 (19.1–29.3)
Deltamethrin	282	90	31.9 (26.5–37.7)
DDT	306	25	8.2 (5.4–11.8)
Fenitrothion	296	219	74.0 (68.6–78.9)
Bendiocarb	299	37	12.4 (8.9–16.6)

*Measured by death within 24 h, after 1h exposure to each insecticide. All mosquitoes were resistant according to World Health Organization classification (<80% dead) (*7*).

To investigate the causes of this resistance, we screened a subset of mosquitoes for the target site mutations, *kdr* 1014F and 1014S. Only the 1014F *kdr* mutation was detected, and this resistance allele was found at high frequency (83%). There was a significant association between presence of the 1014F *kdr* allele and ability to survive exposure to DDT but not to either pyrethroid (Table 2). In contrast, the *Ace-1^R^* allele was strongly associated with survival after exposure to bendiocarb and fenitrothion (Table 2).

**Table 2:** Association between genotype and mosquito survival after insecticide exposure*

Insecticide	No. tested	Status	No.	No. per genotype				Frequency†	Odds ratio§	p value
				LL	LF	FF		1014F‡		
DDT	73	Alive	48	2	7	39		88.5	4	0.02
		Dead	25	2	10	13		72		
Permethrin	88	Alive	44	1	12	31		84.1	1.23	0.82
		Dead	44	3	12	29		79.5		
Deltamethrin	89	Alive	45	1	12	32		84.4	0.82	0.86
		Dead	44	2	9	33		85.2		
				GG	GS	SS		119S¶		
Bendiocarb	86	Alive	49	0	49	0		50	100	0.40 × 10^–12^
		Dead	37	25	12	0		16.2		
Fenitrothion	100	Alive	50	0	50	0		50	1,176	0
		Dead	50	48	2	0		2		

*F and L represent mutant resistant alleles (phenylalanine) and wild-type alleles (leucine), respectively; S and G represent mutant resistant alleles (serine) and wild-type alleles (glycine), respectively. No resistant homozygotes GG were found among the 186 mosquitoes genotyped for *Ace-1^R^* by restriction fragment length polymorphism (a subset of 48 was further screened by using the TaqMan assay; congruence between the 2 methods was 100%). †The frequencies were calculated for each insecticide and mosquito status (alive/dead) after exposure. ‡1014F represent the *kdr* frequencies. §Genotypic odds ratios (ORs) are shown because these exceed allelic ORs for DDT (recessive model), bendiocarb, and fenitrothion (both overdominant models), and are similar for permethrin and deltamethrin. For bendiocarb and fenitrothion absence of GG genotypes in the “Alive” group means that ORs are infinity, therefore ORs are shown if one GG was present. F and L represent mutant resistant alleles (phenylalanine) and wild-type alleles (leucine), respectively; S and G represent mutant resistant alleles (serine) and wild-type alleles. ¶119S represents the *Ace-1^R^* frequencies.

## Conclusions

Pyrethroid resistance in *An. gambiae* mosquitoes was first reported from Côte d’Ivoire in 1993 (*12*); carbamate resistance was detected in the 1990s (*13*). Nevertheless, ≈2 decades later, it is surprising and worrying to find complete resistance to all insecticides tested, particularly—for deltamethrin and bendiocarb—at such high levels. Resistance mechanisms seem to be varied. *Ace-1^R^* is strongly associated with organophosphate and carbamate resistance, and the absence of 119S homozygotes might be attributable to the high fitness cost of the *Ace-1^R^* allele in the absence of insecticide (*14*). Presence of the 1014F *kdr* allele alone does not confer the ability to survive diagnostic doses of pyrethroids; thus, alternative mechanisms must be responsible for the high-level pyrethroid resistance in this population.

The selective pressures responsible for this intense multiple-insecticide resistance in Tiassalé mosquitoes are unclear. There is a high coverage of insecticide-treated bed nets, but this coverage does not differ from that in other parts of the continent, and indoor residual spraying has not been conducted in this region. Use of insecticides in agriculture has been linked to resistance in malaria vectors. This use is perhaps the most likely explanation in this district of intense commercial production of rice, cocoa, and coffee.

Whatever the cause, the implications of this resistance scenario for malaria control are severe. With no new classes of insecticides for malaria control anticipated until 2020 at the earliest (*15*), program managers have few options available when confronted with multiple-insecticide resistance. Assessing the effect of pyrethroid resistance on the efficacy of insecticide-treated bed nets is complex because of the poorly understood associations between net integrity, insecticide content, net usage, and net efficacy. Nevertheless, resistance levels, such as those reported here, combined with continual selection pressure will inevitably lead to suboptimal mosquito control by use of insecticide-treated bed nets and indoor residual spraying. If unchecked, this resistance could spread rapidly and threaten the fragile gains that have been made in reducing malaria across Africa.

Technical Appendix Time-mortality curve for *Anopheles gambiae* mosquitoes, Kisumu strain, exposed to deltamethrin and bendiocarb, and time-death data for adult female *A. gambiae* s.s. mosquitoes, Tiassalé strain, and standard susceptible colony Kisumu 24 hours after exposure to bendiocarb or deltamethrin.
