# Supplementary material for: Multiple-Insecticide Resistance in Anopheles gambiae Mosquitoes, Southern Côte d’Ivoire
Source: Emerg Infect Dis. 2012 Sep;18(9):1508–11. doi: 10.3201/eid1809.120262 (PMC3437712; doi:10.3201/eid1809.120262)
Supplement: Technical Appendix — Time-mortality curve for Anopheles gambiae mosquitoes, Kisumu strain, exposed to deltamethrin and bendiocarb, and time-death data for adult female A. gambiae s.s. mosquitoes, Tiassalé strain, and standard susceptible colony Kisumu 24 hours after exposure to bendiocarb or deltamethrin. [file 12-0262-Techapp-s1.pdf]

Article DOI: http://dx.doi.org/10.3201/eid1809.120262

Multiple-Insecticide Resistance in *Anopheles gambiae* Mosquitoes, Southern Côte d'Ivoire

Technical Appendix

Table**.** Time–death data for adult female *Anopheles gambiae* s.s. Tiassalé strain and standard susceptible colony Kisumu 24 hours after exposure to bendiocarb or deltamethrin

			Exposure time (min)																		
Strain	Insecticides	Parameters	0.5	1	2	3	5	10	15	25	30	60	120	240	260	280	330	340	360	480	720
Tiassalé	Bendiocarb (0.1%)	No. tested										100		100		100	100	100	100	100	
		No. dead										12		28		45	62	67	78	80	
		Mortality (%)										12		28		45	62	67	78	80	
	Deltamethrin (0.05%)	No. tested										100	100	100	100				100	100	100
		No. dead										32	34	49	87				94	95	98
		Mortality (%)										32	34	49	87				94	95	98
Kisumu	Bendiocarb (0.1%)	No. tested					50	50	50	50	50										
		No, dead					2	13	43	48	50										
		Mortality (%)					4	26	86	96	100										
	Deltamethrin (0.05%)	No. tested	50	50	50	50	50	50													
		No. dead	11	23	28	46	49	49													
		Mortality (%)	22	46	56	92	98	98													

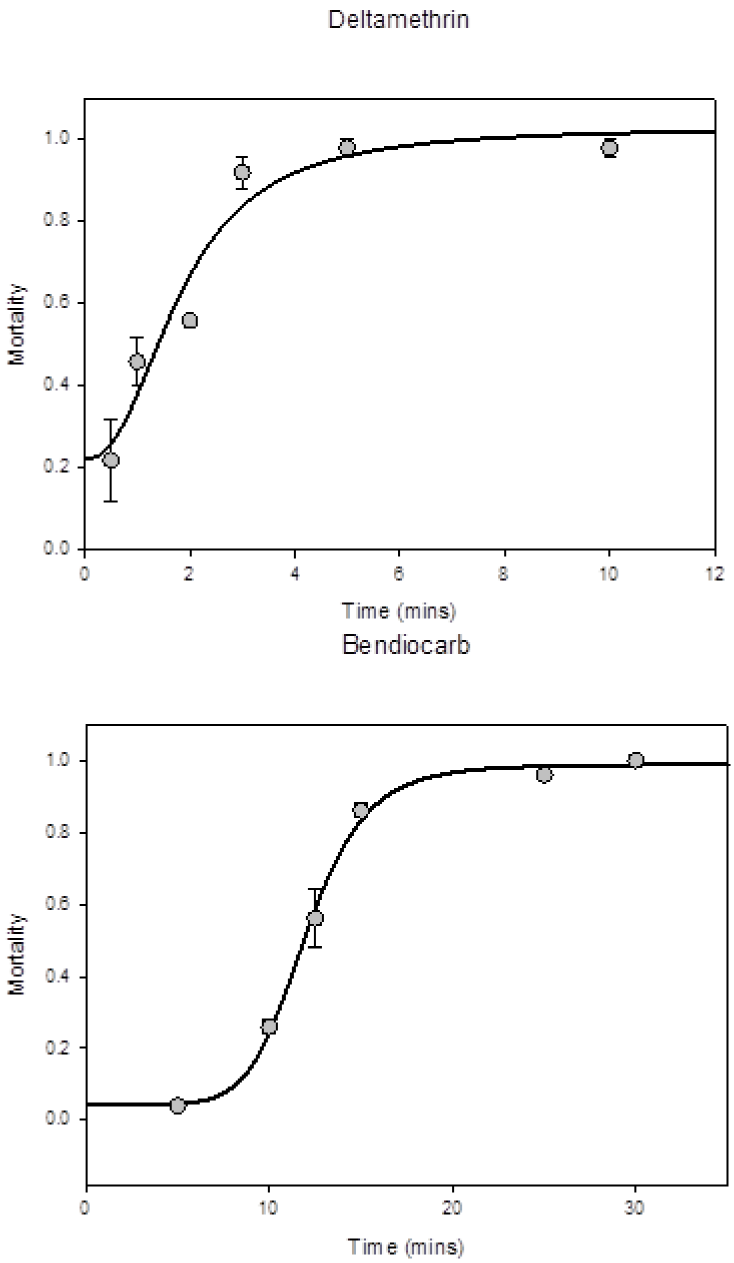

Figure. Time-death curve for Anopheles gambiae mosquitoes, Kisumu strain, exposed to (top) deltamethrin (median time to death = 1.8 minutes, R2= 0.93) and (bottom) bendiocarb (median time to death = 12 minutes, R2= 0.99).
